# Synchronized multiple regression of diagnostic radiation-induced rather than spontaneous: disseminated primary intracranial germinoma in a woman: a case report

**DOI:** 10.1186/1752-1947-5-39

**Published:** 2011-01-27

**Authors:** Yuichiro Yoneoka, Itaru Tsumanuma, Shinya Jinguji, Manabu Natsumeda, Yukihiko Fujii

**Affiliations:** 1 Department of Neurosurgery, Brain Research Institute, University of Niigata 1-757 Asahimachi-dori, Chuo-ku, Niigata, 951-8585, Japan

## Abstract

**Introduction:**

Examples of the spontaneous regression of primary intracranial germinomas can be found in the literature. We present the case of a patient with disseminated lesions of primary intracranial germinoma which synchronously shrunk following diagnostic irradiation. We will discuss whether this regression was spontaneous or radiation-induced.

**Case presentation:**

A 43-year-old Japanese woman presented to our hospital complaining of memory problems over a period of one year and blurred vision over a period of three months. Following magnetic resonance imaging, she was found to have a massive lesion in the third ventricle and small lesions in the pineal region, fourth ventricle, and in the anterior horn of the left lateral ventricle. Prior to an open biopsy to confirm the pathology of the lesions, she underwent a single cranial computed tomography scan and a single cranial digital subtraction angiography for a transcranial biopsy. Fourteen days after the first magnetic resonance image - 12 and eight days after the computed tomography scan and digital subtraction angiography, respectively - a pre-operative magnetic resonance image was taken, which showed a notable synchronous shrinkage of the third ventricle tumor, as well as shrinkage of the lesions in the pineal region and in the fourth ventricle. She did not undergo steroid administration until after a biopsy that confirmed the pathological diagnosis of pure germinoma. She then underwent whole craniospinal irradiation and went into a complete remission.

**Conclusions:**

In our case report, we state that diagnostic radiation can induce the regression of germinomas; this is the most reasonable explanation for the synchronous multiple regression observed in this case of germinoma. Clinicians should keep this non-spontaneous regression in mind and monitor germinoma lesions with minimal exposure to diagnostic radiation before diagnostic confirmation, and also before radiation treatment with or without chemotherapy begins.

## Introduction

Spontaneous regression and remission from cancer was defined by Cole and Everson in 1956 [1]. Examples of the spontaneous regression of primary intracranial germinomas can be found in the literature [2-5]. In our case report, we present the case of a patient with disseminated lesions of primary intracranial germinoma who experienced synchronous shrinkage of disseminated lesions of germinoma following diagnostic irradiation. We discuss whether this regression was spontaneous or diagnostic radiation-induced.

### Case presentation

**Figure 1:**
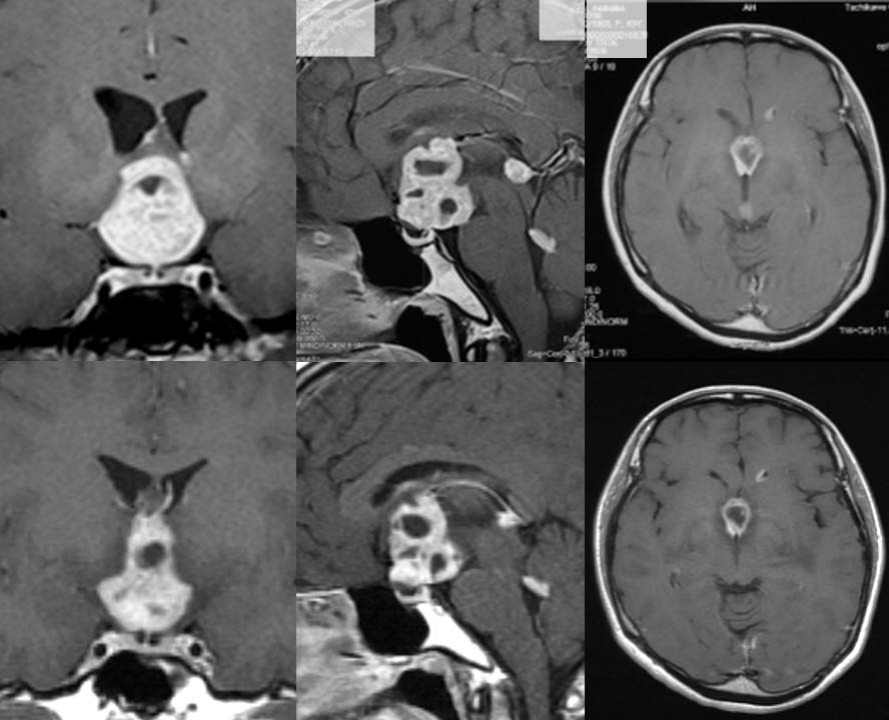
**Contrast-enhanced MRI showing multiple enhanced lesions in the third ventricle, pineal region, and fourth ventricle (upper)**.

**Figure 2:**
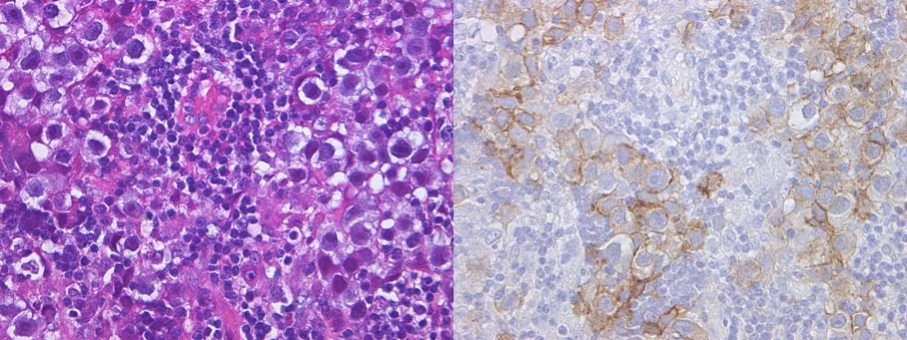
**Pathological findings: A hematoxylin and eosin stain demonstrating a biphasic cell population of lymphocytes with admixed large cells (left), in which a placental alkaline phosphatase stain highlights the germinoma cells (right)**.

**Figure 3:**
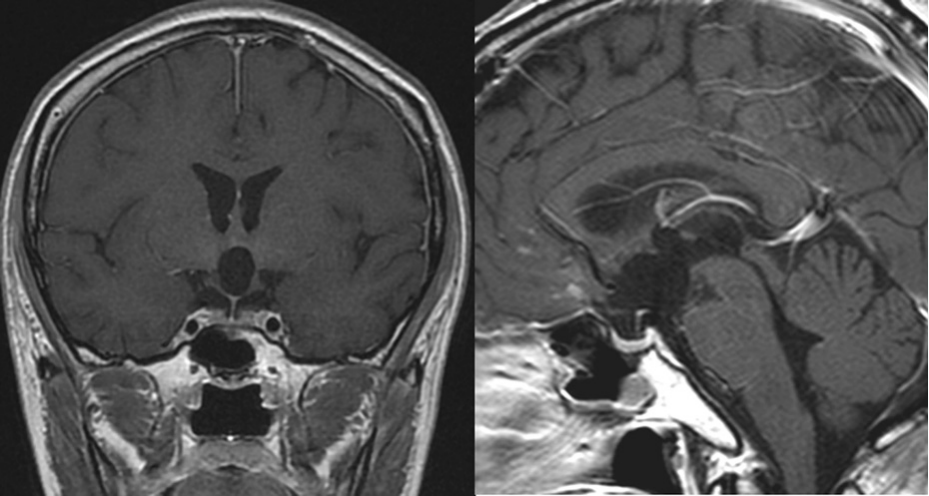
**Post-radiotherapy MRI demonstrating a complete remission of the germinoma lesions**.

A 43-year-old Japanese woman presented to our hospital complaining of memory problems over a period of one year and blurred vision over a period of three months. Following magnetic resonance imaging (MRI), she was found to have a massive lesion in the third ventricle and small lesions in the pineal region, fourth ventricle, and in the anterior horn of the left lateral ventricle (Figure 1). A blood examination revealed hypothyroidism, and she received hormone replacement therapy with levothyroxine (50 micrograms per day for two days) until her biopsy. Before a second MRI to confirm the pathology of the lesions, she underwent a single computed tomography (CT) scan and a single cranial digital subtraction angiography (DSA). Fourteen days after the first MRI - 12 and eight days after the CT scan and DSA, respectively - a pre-operative MRI was taken, which showed a notable synchronous shrinkage of the third ventricle tumor, as well as shrinkage of the lesions in the pineal region and in the fourth ventricle. We did not administer steroids until an open biopsy pathologically diagnosed the tumor as a pure germinoma. She underwent radiation treatment to the whole brain and spine after confirmation of the pathological diagnosis (Figure 2). Although she suffered memory impairment, she went into a complete remission following the radiation therapy (Figure 3).

## Discussion

After Ide *et al. *reported the first suspected case of the regression of a primary intracranial germinoma [3], several reports about the regression of primary intracranial germinomas were added to the literature [1-5]. The patients in these cases received diagnostic radiation treatments, such as a cranial CT scan and/or angiography, without any known exceptions [2-5]. As is evident from the literature, germinomas have high radiosensitivity [6]. It is possible to shrink a germinoma with a single craniogram. In fact, shrunken germinomas demonstrate regrowth if carefully monitored [3,5]. Based on our experience of diagnostic irradiation and/or radiotherapy for the treatment of germinomas, and through our review of the literature about the regression of germinomas [2-5], we have speculated that the shrinkage of the lesions in our case report was not a "spontaneous" regression but rather a diagnostic radiation-induced regression.

A second MRI, at 12 and eight days after the CT scan and DSA, respectively, confirmed the synchronous regression of the disseminated germinoma. Prior to confirmation of the synchronous regression, it was estimated that the patient had received a dose of diagnostic radiation of 27.0-41.4mGy (0.3-0.4Gy rounded). This figure was obtained as the sum of 23.4-37.8mGy (2.34-3.78cGy in the original text [7]) of a single cranial CT scan [7] and 3.6mGy of a single DSA (3.6mSv in the original text; Sv = Gy in X-ray) [8].

Germinomas are so radiosensitive that they occasionally show regression after exposure to the radiation for diagnostic angiography [9]. This shrinkage by diagnostic angiography enhances the radiosensitive nature of germinomas and supports the suggestion that the synchronous regression observed in our case report was not spontaneous but rather diagnostic radiation-induced.

Moreover, a correlative pathologic and imaging (CT and MRI) study reports the case of a 35-year-old man with a pineal germinoma who died unexpectedly of a massive pulmonary embolism on the eighth day of a course of radiation therapy after receiving a total dose of only 16Gy ( = 2Gy/day × 8 times). A histological study of the entire lesion in the serial sections of pathological specimen revealed no viable tumor cells [6]. This report suggests that the highly radiosensitive nature of germinomas can result in a synchronous multiple regression of disseminated germinomas as a result of low-dose radiation.

Germinomas tend to be treated with a lower dose of radiation applied to a smaller volume of exposure field than those used with conventional radiotherapy of 40-55Gy [10], using fractionated radiation therapy with a fraction size of <2.5Gy. The estimated dose of diagnostic radiation received by our patient before the regression of the germinoma was 0.3-0.4Gy, which is smaller than the fraction size of radiation therapy for germinomas ( = 2Gy).

This leads us to question whether previously-reported spontaneous regressions of germinomas really are "spontaneous". Significant percentages of previously-reported cases of the "spontaneous" regression of germinomas probably include radiation-induced regression, because the patients in all of these cases were exposed to diagnostic radiation; for example, plain X-ray films [2], CT scan(s) [3-5], and angiography [2].

The periods from diagnostic irradiation to the detection of a regression in these cases from the literature are summarized in Table 1. The diagnostic radiation-induced regression of germinomas was observed between six and 56 days after diagnostic radiation (Table 1). This interval between diagnostic radiation and the regression of the tumors is a key point. To the best of our knowledge, no germinoma regressions have been reported in patients who had not previously undergone diagnostic irradiation.

**Table 1:** Summary of cases demonstrating regression of intracranial germinomas

Author	Age/Sex	Lesion(s)	Size before regression	Operation before regression	Steroid before regression	Diagnostic radiation before regression	Period from diagnostic radiation to detection of regression	Involution period of tumor
Ide *et al. *[3]	21/M	Neurophypophysis	Larger than 20 mm	VP shunt	+	CT	6 days	2 months
Fujimaki *et al. *[2]	39/M	Pineal, IV ventricle	Larger than 20 mm	Tumor removal	+	X-ray film, cerebral angiography	15 days from X-ray film, 6 days from angiography	N/A
Murai *et al. *[4]	17/M	Pineal	30 mm	VP shunt	none	7 CTs	56 days from 7th CT	N/A
Sato *et al. *[5]	13/M	Neurophypophysis, Pineal	13 mm, 20 mm	none	none	CT	13 days	3 weeks
Our case report	43/F	Neurophypophysis, Pineal, Llateral ventricle, IV ventricle	32 mm, 10 mm, 6 mm, 9 mm	none	none	CT, cerebral angiography	12 days from CT, 8 days from angiography	N/A

Si *et al. *reported the case of a patient with a central nervous system germinoma that showed a significant regression in size following surgery and the administration of dexamethasone, prior to the initiation of chemotherapy or irradiation [11]. However, the patient underwent multiple cranial CT scans so, even in this case, we cannot be certain that the regression is not also diagnostic radiation-induced.

## Conclusions

Clinicians should keep in mind that diagnostic radiation can induce the regression of intracranial germinomas and they should monitor germinoma lesions with minimal exposure to diagnostic radiation before diagnostic confirmation, and also before radiation treatment with/without chemotherapy begins. Regressions induced by diagnostic radiation may also indicate the high radiosensitivity of the lesion, which is key to an accurate diagnosis of germinoma. This provides a diagnostic and/or therapeutic clue and can help avoid radical resection.

## Competing interests

The authors declare that they have no competing interests.

## Consent

Written informed consent was obtained from the patient for publication of this case report and any accompanying images. A copy of the written consent is available for review by the Editor-in-Chief of this journal.

## Authors' contributions

YY and IT collected the clinical data and drafted the manuscript. YY analyzed and interpreted the patient data regarding the germinoma and its radiosensitivity. SJ reviewed the literature. MN reported on the histopathological specimen. YF critically revised the manuscript. All authors read and approved the final manuscript.
